# Hexaaqua­magnesium(II) bis­[4-(3-eth­oxy-2-hydroxy­benzyl­ideneamino)-3-methyl­benzene­sulfonate]

**DOI:** 10.1107/S1600536810009748

**Published:** 2010-03-20

**Authors:** Xi-Shi Tai, Fu-Gong Zhang

**Affiliations:** a Department of Chemistry, Weifang University, Weifang 261061, People’s Republic of China; b Department of Physics, Weifang University, Weifang 261061, People’s Republic of China

## Abstract

In the title compound, [Mg(H_2_O)_6_](C_16_H_16_NO_5_S)_2_, the Mg^2+^ ion (site symmetry 2) adopts an almost regular octa­hedral coordination geometry. The anion is stabilized by an intra­molecular O—H⋯N hydrogen bond, generating an *S*(6) ring, and the dihedral angle between the aromatic rings is 41.02 (7)°. In the crystal, the cations and anions are linked by O—H⋯O hydrogen bonds, generating sheets lying parallel to (100).

## Related literature

For background to the properties of Schiff bases, see: Qiu *et al.* (2008 ▶); Tai *et al.* (2003 ▶). 
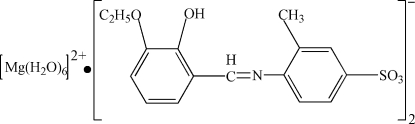


## Experimental

### 

#### Crystal data

[Mg(H_2_O)_6_](C_16_H_16_NO_5_S)
*M* *_r_* = 801.12
Monoclinic, 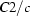

*a* = 38.710 (11) Å
*b* = 7.531 (2) Å
*c* = 13.087 (3) Å
β = 104.986 (4)°
*V* = 3685.6 (17) Å^3^
*Z* = 4
Mo *K*α radiation
μ = 0.24 mm^−1^
*T* = 293 K
0.19 × 0.16 × 0.12 mm

#### Data collection

Bruker SMART CCD diffractometer
Absorption correction: multi-scan (*SADABS*; Bruker, 2000 ▶) *T*_min_ = 0.956, *T*_max_ = 0.972
9339 measured reflections
3253 independent reflections
2924 reflections with *I* > 2σ(*I*)
*R*_int_ = 0.024

#### Refinement

*R*[*F* ^2^ > 2σ(*F* ^2^)] = 0.032
*wR*(*F* ^2^) = 0.088
*S* = 1.07
3253 reflections
241 parameters
H-atom parameters constrained
Δρ_max_ = 0.21 e Å^−3^
Δρ_min_ = −0.48 e Å^−3^

### 

Data collection: *SMART* (Bruker, 2000 ▶); cell refinement: *SAINT* (Bruker, 2000 ▶); data reduction: *SAINT*; program(s) used to solve structure: *SHELXS97* (Sheldrick, 2008 ▶); program(s) used to refine structure: *SHELXL97* (Sheldrick, 2008 ▶); molecular graphics: *SHELXTL* (Sheldrick, 2008 ▶); software used to prepare material for publication: *SHELXTL*.

## Supplementary Material

Crystal structure: contains datablocks global, I. DOI: 10.1107/S1600536810009748/hb5354sup1.cif

Structure factors: contains datablocks I. DOI: 10.1107/S1600536810009748/hb5354Isup2.hkl

Additional supplementary materials:  crystallographic information; 3D view; checkCIF report

## Figures and Tables

**Table 1:** Selected bond lengths (Å)

Mg1—O6	2.0510 (11)
Mg1—O8	2.0605 (12)
Mg1—O7	2.0638 (12)

**Table 2:** Hydrogen-bond geometry (Å, °)

*D*—H⋯*A*	*D*—H	H⋯*A*	*D*⋯*A*	*D*—H⋯*A*
O4—H4⋯N1	0.82	1.91	2.6355 (18)	146
O6—H15⋯O2^i^	0.85	2.04	2.8744 (16)	168
O6—H16⋯O1^ii^	0.85	2.01	2.8416 (16)	165
O7—H17⋯O1^iii^	0.85	2.00	2.8349 (17)	166
O7—H18⋯O3^ii^	0.85	2.02	2.8528 (16)	166
O8—H19⋯O3^i^	0.85	2.02	2.8563 (16)	168
O8—H20⋯O2^iii^	0.85	2.08	2.8895 (17)	159

Symmetry codes: (i) 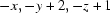; (ii) 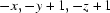; (iii) 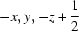.

**Fig. 1.**
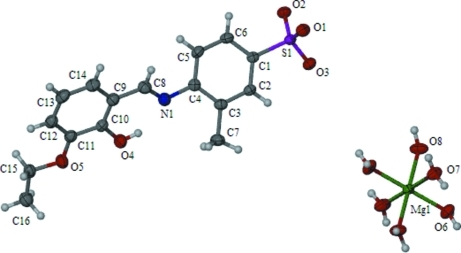
The molecular structure of (I) showing 30% displacement ellipsoids.

## supplementary crystallographic information

### Comment

Schiff bases play an important role in the field of bioinorganic chemistry because they have remarkable wide biological and pharmacological activities, such as antitumor, antidiabetic, antitubercular activities [Tai, *et al.*, 2003; Qiu, *et al.*, 2008]. Therefore, investigating the synthesis and proper ties of hydrazone of these compounds seems to be a very interesting problem. as one part of our systematic work, In this paper, we report on the synthesis and crystal structure of the title compound, (I), (Scheme I).

The bond distances of Mg—O are in the range of 2.0510 (11)-2.0638 (12). The bond distances of C8—N1(1.282 (2)), S1—O2 (1.4588 (11)) and S1—O3 (1.4590 (11)) are consistent with the carbon-nitrogen and sulphur-oxygen double-bond lengths, respectively. In the crystal packing, the molecules form a one-dimensional chain structure by hydrogen bonds.

### Experimental

A solution of 1.0 mmol 3-ethoxysalicylaldehyde was added to a solution of 1.0 mmol 4-amino-3-methyl-benzenesulfonic acid in 5 ml 95% ethanol at room temperature. The mixture was refluxed for 4 h with stirring, then the resulting precipitate was filtered, washed, and dried in vacuo over P_4_O_10_ for 48 h.  Colourless blocks of (I) were obtained by slowly evaporating from methanol at room temperature.

### Crystal data

**Table:**

[Mg(H_2_O)_6_](C_16_H_16_NO_5_S)	*F*(000) = 1688
*M**_r_* = 801.12	*D*_x_ = 1.444 Mg m^−^^3^
Monoclinic, *C*2/*c*	Mo *K*α radiation, λ = 0.71073 Å
Hall symbol: -C 2yc	Cell parameters from 5727 reflections
*a* = 38.710 (11) Å	θ = 3.1–28.3°
*b* = 7.531 (2) Å	µ = 0.24 mm^−^^1^
*c* = 13.087 (3) Å	*T* = 293 K
β = 104.986 (4)°	Block, colourless
*V* = 3685.6 (17) Å^3^	0.19 × 0.16 × 0.12 mm
*Z* = 4	

### Data collection

**Table:**

Bruker SMART CCD diffractometer	3253 independent reflections
Radiation source: fine-focus sealed tube	2924 reflections with *I* > 2σ(*I*)
graphite	*R*_int_ = 0.024
ω scans	θ_max_ = 25.1°, θ_min_ = 2.2°
Absorption correction: multi-scan (*SADABS*; Bruker, 2000)	*h* = −46→41
*T*_min_ = 0.956, *T*_max_ = 0.972	*k* = −8→8
9339 measured reflections	*l* = −15→15

### Refinement

**Table:**

Refinement on *F*^2^	Primary atom site location: structure-invariant direct methods
Least-squares matrix: full	Secondary atom site location: difference Fourier map
*R*[*F*^2^ > 2σ(*F*^2^)] = 0.032	Hydrogen site location: inferred from neighbouring sites
*wR*(*F*^2^) = 0.088	H-atom parameters constrained
*S* = 1.07	*w* = 1/[σ^2^(*F*_o_^2^) + (0.0466*P*)^2^ + 2.1281*P*] where *P* = (*F*_o_^2^ + 2*F*_c_^2^)/3
3253 reflections	(Δ/σ)_max_ < 0.001
241 parameters	Δρ_max_ = 0.21 e Å^−^^3^
0 restraints	Δρ_min_ = −0.48 e Å^−^^3^

### Special details

**Table:**

Geometry. All esds (except the esd in the dihedral angle between two l.s. planes) are estimated using the full covariance matrix. The cell esds are taken into account individually in the estimation of esds in distances, angles and torsion angles; correlations between esds in cell parameters are only used when they are defined by crystal symmetry. An approximate (isotropic) treatment of cell esds is used for estimating esds involving l.s. planes.
Refinement. Refinement of *F*^2^ against ALL reflections. The weighted *R*-factor wR and goodness of fit *S* are based on *F*^2^, conventional *R*-factors *R* are based on *F*, with *F* set to zero for negative *F*^2^. The threshold expression of *F*^2^ > σ(*F*^2^) is used only for calculating *R*-factors(gt) etc. and is not relevant to the choice of reflections for refinement. *R*-factors based on *F*^2^ are statistically about twice as large as those based on *F*, and *R*- factors based on ALL data will be even larger.

### Fractional atomic coordinates and isotropic or equivalent isotropic displacement parameters (Å^2^)

**Table:**

	*x*	*y*	*z*	*U*_iso_*/*U*_eq_	
Mg1	0.0000	0.76200 (8)	0.7500	0.02561 (17)	
S1	0.053241 (9)	0.73171 (5)	0.12108 (3)	0.02690 (12)	
N1	0.21154 (3)	0.64726 (18)	0.21861 (10)	0.0358 (3)	
O1	0.03738 (3)	0.57836 (14)	0.05783 (8)	0.0372 (3)	
O2	0.04508 (3)	0.89760 (14)	0.06222 (8)	0.0357 (3)	
O3	0.04451 (3)	0.73756 (14)	0.22300 (8)	0.0352 (3)	
O4	0.27591 (3)	0.69109 (17)	0.34589 (8)	0.0414 (3)	
H4	0.2540	0.6876	0.3293	0.062*	
O5	0.34554 (3)	0.68788 (17)	0.37345 (9)	0.0426 (3)	
O6	−0.03010 (3)	0.76330 (14)	0.85798 (9)	0.0404 (3)	
H15	−0.0333	0.8568	0.8908	0.061*	
H16	−0.0320	0.6714	0.8938	0.061*	
O7	−0.03189 (3)	0.56722 (15)	0.66233 (8)	0.0409 (3)	
H17	−0.0329	0.5509	0.5974	0.061*	
H18	−0.0353	0.4662	0.6870	0.061*	
O8	−0.03191 (3)	0.95651 (15)	0.66277 (8)	0.0458 (3)	
H19	−0.0368	1.0546	0.6880	0.069*	
H20	−0.0359	0.9680	0.5961	0.069*	
C1	0.10021 (4)	0.70263 (19)	0.14849 (11)	0.0280 (3)	
C2	0.11930 (4)	0.63608 (19)	0.24591 (11)	0.0309 (3)	
H2	0.1071	0.6055	0.2960	0.037*	
C3	0.15615 (4)	0.6144 (2)	0.26994 (11)	0.0320 (3)	
C4	0.17378 (4)	0.6597 (2)	0.19257 (12)	0.0318 (3)	
C5	0.15439 (4)	0.7242 (2)	0.09457 (12)	0.0356 (4)	
H5	0.1663	0.7536	0.0437	0.043*	
C6	0.11778 (4)	0.7452 (2)	0.07202 (12)	0.0340 (4)	
H6	0.1051	0.7875	0.0063	0.041*	
C7	0.17670 (4)	0.5461 (2)	0.37649 (12)	0.0431 (4)	
H7A	0.1902	0.4432	0.3672	0.065*	
H7B	0.1603	0.5148	0.4174	0.065*	
H7C	0.1927	0.6367	0.4127	0.065*	
C8	0.22688 (4)	0.5956 (2)	0.14795 (13)	0.0380 (4)	
H8	0.2127	0.5609	0.0823	0.046*	
C9	0.26537 (4)	0.5886 (2)	0.16581 (12)	0.0353 (3)	
C10	0.28823 (4)	0.6380 (2)	0.26306 (12)	0.0327 (3)	
C11	0.32543 (4)	0.6332 (2)	0.27646 (12)	0.0343 (3)	
C12	0.33903 (4)	0.5734 (2)	0.19455 (13)	0.0387 (4)	
H12	0.3636	0.5698	0.2035	0.046*	
C13	0.31630 (5)	0.5186 (2)	0.09917 (13)	0.0417 (4)	
H13	0.3258	0.4756	0.0455	0.050*	
C14	0.27993 (5)	0.5279 (2)	0.08417 (13)	0.0414 (4)	
H14	0.2648	0.4939	0.0197	0.050*	
C15	0.38322 (4)	0.7012 (2)	0.38726 (13)	0.0384 (4)	
H15A	0.3885	0.7889	0.3392	0.046*	
H15B	0.3929	0.5878	0.3729	0.046*	
C16	0.39945 (5)	0.7556 (2)	0.49975 (15)	0.0470 (4)	
H16A	0.3908	0.8712	0.5118	0.070*	
H16B	0.4250	0.7590	0.5129	0.070*	
H16C	0.3929	0.6714	0.5466	0.070*	

### Atomic displacement parameters (Å^2^)

**Table:**

	*U*^11^	*U*^22^	*U*^33^	*U*^12^	*U*^13^	*U*^23^
Mg1	0.0316 (4)	0.0244 (3)	0.0216 (3)	0.000	0.0083 (3)	0.000
S1	0.0321 (2)	0.0256 (2)	0.0227 (2)	−0.00147 (13)	0.00662 (15)	−0.00049 (13)
N1	0.0347 (7)	0.0373 (7)	0.0357 (7)	−0.0021 (6)	0.0095 (6)	−0.0026 (6)
O1	0.0446 (6)	0.0337 (6)	0.0315 (6)	−0.0093 (5)	0.0067 (5)	−0.0049 (5)
O2	0.0427 (6)	0.0314 (6)	0.0324 (6)	0.0033 (5)	0.0087 (5)	0.0052 (4)
O3	0.0422 (6)	0.0378 (6)	0.0285 (6)	0.0008 (5)	0.0143 (5)	−0.0002 (4)
O4	0.0356 (6)	0.0564 (7)	0.0342 (6)	0.0017 (6)	0.0126 (5)	−0.0072 (5)
O5	0.0323 (6)	0.0569 (7)	0.0391 (6)	0.0010 (5)	0.0098 (5)	−0.0048 (6)
O6	0.0551 (7)	0.0334 (6)	0.0411 (7)	0.0028 (5)	0.0277 (6)	0.0015 (5)
O7	0.0572 (7)	0.0356 (6)	0.0294 (5)	−0.0140 (5)	0.0103 (5)	−0.0040 (5)
O8	0.0668 (8)	0.0374 (6)	0.0299 (6)	0.0180 (6)	0.0067 (5)	0.0026 (5)
C1	0.0334 (8)	0.0251 (7)	0.0251 (7)	−0.0015 (6)	0.0066 (6)	−0.0025 (6)
C2	0.0373 (8)	0.0315 (8)	0.0251 (7)	−0.0013 (6)	0.0099 (6)	0.0006 (6)
C3	0.0380 (8)	0.0291 (8)	0.0274 (7)	0.0004 (6)	0.0059 (6)	−0.0010 (6)
C4	0.0343 (8)	0.0284 (8)	0.0329 (8)	−0.0009 (6)	0.0088 (6)	−0.0038 (6)
C5	0.0412 (9)	0.0383 (9)	0.0309 (8)	0.0005 (7)	0.0158 (7)	0.0031 (6)
C6	0.0415 (9)	0.0346 (8)	0.0258 (8)	0.0037 (6)	0.0084 (6)	0.0035 (6)
C7	0.0425 (9)	0.0536 (11)	0.0316 (8)	0.0066 (8)	0.0067 (7)	0.0057 (8)
C8	0.0399 (9)	0.0393 (9)	0.0337 (8)	−0.0004 (7)	0.0077 (7)	−0.0044 (7)
C9	0.0383 (8)	0.0344 (8)	0.0346 (8)	0.0009 (7)	0.0119 (6)	−0.0007 (7)
C10	0.0380 (8)	0.0298 (8)	0.0328 (8)	0.0012 (6)	0.0138 (6)	0.0011 (6)
C11	0.0363 (8)	0.0323 (8)	0.0355 (8)	0.0033 (6)	0.0116 (6)	0.0042 (6)
C12	0.0398 (9)	0.0378 (9)	0.0425 (9)	0.0054 (7)	0.0178 (7)	0.0057 (7)
C13	0.0528 (10)	0.0403 (9)	0.0388 (9)	0.0064 (8)	0.0241 (8)	0.0011 (7)
C14	0.0491 (10)	0.0440 (10)	0.0326 (8)	0.0011 (8)	0.0132 (7)	−0.0031 (7)
C15	0.0335 (8)	0.0371 (9)	0.0458 (9)	0.0024 (7)	0.0124 (7)	0.0042 (7)
C16	0.0405 (10)	0.0506 (11)	0.0481 (11)	−0.0012 (7)	0.0083 (8)	−0.0010 (8)

### Geometric parameters (Å, °)

**Table:**

Mg1—O6^i^	2.0510 (11)	C3—C4	1.402 (2)
Mg1—O6	2.0510 (11)	C3—C7	1.506 (2)
Mg1—O8^i^	2.0605 (12)	C4—C5	1.395 (2)
Mg1—O8	2.0605 (12)	C5—C6	1.380 (2)
Mg1—O7	2.0638 (12)	C5—H5	0.9300
Mg1—O7^i^	2.0638 (12)	C6—H6	0.9300
S1—O2	1.4588 (11)	C7—H7A	0.9600
S1—O3	1.4590 (11)	C7—H7B	0.9600
S1—O1	1.4605 (11)	C7—H7C	0.9600
S1—C1	1.7735 (16)	C8—C9	1.448 (2)
N1—C8	1.282 (2)	C8—H8	0.9300
N1—C4	1.416 (2)	C9—C10	1.399 (2)
O4—C10	1.3526 (18)	C9—C14	1.407 (2)
O4—H4	0.8200	C10—C11	1.405 (2)
O5—C11	1.3695 (19)	C11—C12	1.386 (2)
O5—C15	1.4262 (19)	C12—C13	1.391 (2)
O6—H15	0.8499	C12—H12	0.9300
O6—H16	0.8499	C13—C14	1.372 (2)
O7—H17	0.8500	C13—H13	0.9300
O7—H18	0.8500	C14—H14	0.9300
O8—H19	0.8497	C15—C16	1.500 (2)
O8—H20	0.8498	C15—H15A	0.9700
C1—C6	1.385 (2)	C15—H15B	0.9700
C1—C2	1.391 (2)	C16—H16A	0.9600
C2—C3	1.389 (2)	C16—H16B	0.9600
C2—H2	0.9300	C16—H16C	0.9600
			
O6^i^—Mg1—O6	179.45 (7)	C6—C5—C4	121.00 (14)
O6^i^—Mg1—O8^i^	90.71 (5)	C6—C5—H5	119.5
O6—Mg1—O8^i^	88.90 (5)	C4—C5—H5	119.5
O6^i^—Mg1—O8	88.90 (5)	C5—C6—C1	119.21 (14)
O6—Mg1—O8	90.71 (5)	C5—C6—H6	120.4
O8^i^—Mg1—O8	89.38 (7)	C1—C6—H6	120.4
O6^i^—Mg1—O7	89.14 (5)	C3—C7—H7A	109.5
O6—Mg1—O7	91.25 (5)	C3—C7—H7B	109.5
O8^i^—Mg1—O7	179.85 (5)	H7A—C7—H7B	109.5
O8—Mg1—O7	90.61 (5)	C3—C7—H7C	109.5
O6^i^—Mg1—O7^i^	91.25 (5)	H7A—C7—H7C	109.5
O6—Mg1—O7^i^	89.14 (5)	H7B—C7—H7C	109.5
O8^i^—Mg1—O7^i^	90.61 (5)	N1—C8—C9	122.93 (15)
O8—Mg1—O7^i^	179.85 (5)	N1—C8—H8	118.5
O7—Mg1—O7^i^	89.40 (7)	C9—C8—H8	118.5
O2—S1—O3	112.78 (6)	C10—C9—C14	119.59 (15)
O2—S1—O1	112.09 (7)	C10—C9—C8	121.29 (14)
O3—S1—O1	112.28 (6)	C14—C9—C8	119.11 (14)
O2—S1—C1	106.45 (7)	O4—C10—C9	122.43 (14)
O3—S1—C1	106.66 (7)	O4—C10—C11	118.02 (13)
O1—S1—C1	106.00 (7)	C9—C10—C11	119.55 (14)
C8—N1—C4	119.24 (13)	O5—C11—C12	125.18 (14)
C10—O4—H4	109.5	O5—C11—C10	115.24 (13)
C11—O5—C15	117.14 (12)	C12—C11—C10	119.57 (14)
Mg1—O6—H15	122.3	C11—C12—C13	120.80 (15)
Mg1—O6—H16	121.5	C11—C12—H12	119.6
H15—O6—H16	110.6	C13—C12—H12	119.6
Mg1—O7—H17	121.6	C14—C13—C12	120.05 (15)
Mg1—O7—H18	123.7	C14—C13—H13	120.0
H17—O7—H18	106.2	C12—C13—H13	120.0
Mg1—O8—H19	124.4	C13—C14—C9	120.37 (15)
Mg1—O8—H20	124.3	C13—C14—H14	119.8
H19—O8—H20	108.1	C9—C14—H14	119.8
C6—C1—C2	120.12 (14)	O5—C15—C16	107.37 (14)
C6—C1—S1	119.63 (11)	O5—C15—H15A	110.2
C2—C1—S1	120.24 (11)	C16—C15—H15A	110.2
C3—C2—C1	121.40 (14)	O5—C15—H15B	110.2
C3—C2—H2	119.3	C16—C15—H15B	110.2
C1—C2—H2	119.3	H15A—C15—H15B	108.5
C2—C3—C4	118.12 (13)	C15—C16—H16A	109.5
C2—C3—C7	121.04 (14)	C15—C16—H16B	109.5
C4—C3—C7	120.84 (14)	H16A—C16—H16B	109.5
C5—C4—C3	120.14 (14)	C15—C16—H16C	109.5
C5—C4—N1	121.40 (14)	H16A—C16—H16C	109.5
C3—C4—N1	118.38 (13)	H16B—C16—H16C	109.5
			
O2—S1—C1—C6	−39.83 (13)	C4—N1—C8—C9	176.38 (14)
O3—S1—C1—C6	−160.48 (12)	N1—C8—C9—C10	0.3 (3)
O1—S1—C1—C6	79.69 (13)	N1—C8—C9—C14	179.19 (16)
O2—S1—C1—C2	140.68 (12)	C14—C9—C10—O4	−177.47 (15)
O3—S1—C1—C2	20.03 (14)	C8—C9—C10—O4	1.4 (2)
O1—S1—C1—C2	−99.81 (13)	C14—C9—C10—C11	2.6 (2)
C6—C1—C2—C3	1.4 (2)	C8—C9—C10—C11	−178.57 (14)
S1—C1—C2—C3	−179.08 (11)	C15—O5—C11—C12	6.7 (2)
C1—C2—C3—C4	−0.7 (2)	C15—O5—C11—C10	−174.24 (13)
C1—C2—C3—C7	178.62 (15)	O4—C10—C11—O5	−1.4 (2)
C2—C3—C4—C5	−0.1 (2)	C9—C10—C11—O5	178.52 (14)
C7—C3—C4—C5	−179.45 (15)	O4—C10—C11—C12	177.67 (14)
C2—C3—C4—N1	176.55 (13)	C9—C10—C11—C12	−2.4 (2)
C7—C3—C4—N1	−2.8 (2)	O5—C11—C12—C13	179.20 (15)
C8—N1—C4—C5	−39.8 (2)	C10—C11—C12—C13	0.2 (2)
C8—N1—C4—C3	143.58 (16)	C11—C12—C13—C14	1.8 (3)
C3—C4—C5—C6	0.2 (2)	C12—C13—C14—C9	−1.6 (3)
N1—C4—C5—C6	−176.35 (14)	C10—C9—C14—C13	−0.6 (2)
C4—C5—C6—C1	0.5 (2)	C8—C9—C14—C13	−179.48 (15)
C2—C1—C6—C5	−1.3 (2)	C11—O5—C15—C16	−177.74 (14)
S1—C1—C6—C5	179.20 (11)		

Symmetry codes: (i) −*x*, *y*, −*z*+3/2.

### Hydrogen-bond geometry (Å, °)

**Table:**

*D*—H···*A*	*D*—H	H···*A*	*D*···*A*	*D*—H···*A*
O4—H4···N1	0.82	1.91	2.6355 (18)	146
O6—H15···O2^ii^	0.85	2.04	2.8744 (16)	168
O6—H16···O1^iii^	0.85	2.01	2.8416 (16)	165
O7—H17···O1^iv^	0.85	2.00	2.8349 (17)	166
O7—H18···O3^iii^	0.85	2.02	2.8528 (16)	166
O8—H19···O3^ii^	0.85	2.02	2.8563 (16)	168
O8—H20···O2^iv^	0.85	2.08	2.8895 (17)	159

Symmetry codes: (ii) −*x*, −*y*+2, −*z*+1; (iii) −*x*, −*y*+1, −*z*+1; (iv) −*x*, *y*, −*z*+1/2.
